# Increased Circulating Cytokines Have a Role in COVID-19 Severity and Death With a More Pronounced Effect in Males: A Systematic Review and Meta-Analysis

**DOI:** 10.3389/fphar.2022.802228

**Published:** 2022-02-14

**Authors:** Huating Hu, Hudan Pan, Runze Li, Kancheng He, Han Zhang, Liang Liu

**Affiliations:** ^1^ State Key Laboratory of Quality Research in Chinese Medicine, Macau Institute for Applied Research in Medicine and Health, Macau University of Science and Technology, Macao, China; ^2^ Dr. Neher’s Biophysics Laboratory for Innovative Drug Discovery, Macau University of Science and Technology, Macao, China; ^3^ Department of Urology, The Fifth Affiliated Hospital of Sun Yat-Sen University, Zhuhai, China; ^4^ Institute of Traditional Chinese Medicine Research, Tianjin University of Traditional Chinese Medicine, Zhuhai, China

**Keywords:** COVID-19, cytokines, sex bias, mortality, meta-analysis

## Abstract

**Background:** Coronavirus disease 2019 (COVID-2019), caused by severe acute respiratory syndrome coronavirus 2 (SARS-CoV-2), has become a worldwide epidemic and claimed millions of lives. Accumulating evidence suggests that cytokines storms are closely associated to COVID-19 severity and death. Here, we aimed to explore the key factors related to COVID-19 severity and death, especially in terms of the male patients and those in western countries.

**Methods:** To clarify whether inflammatory cytokines have role in COVID-19 severity and death, we systematically searched PubMed, Embase, Cochrane library and Web of Science to identify related studies with the keywords “COVID-19″ and “cytokines”. The data were measured as the mean with 95% confidence interval (CI) by Review Manager 5.3 software. The risk of bias was assessed for each study using appropriate checklists.

**Results:** We preliminarily screened 13,468 studies from the databases. A total of 77 articles with 13,468 patients were ultimately included in our study. The serum levels of cytokines such as interleukin-6 (IL-6), IL-10, interleukin-2 receptor (IL-2R), tumor necrosis factor (TNF)-α, IL-1β, IL-4, IL-8 and IL-17 were higher in the severity or death group. Notably, we also found that the circulating levels of IL-6, IL-10, IL-2R and TNF-α were significantly different between males and females. The serum levels of IL-6, IL-10, IL-2R and TNF-α were much higher in males than in females, which implies that the increased mortality and severity in males was partly due to the higher level of these cytokines. Moreover, we found that in the severe and non-survivor groups, European patients had elevated levels of IL-6 compared with Asian patients.

**Conclusion:** These large-scale data demonstrated that the circulating levels of IL-6, IL-10, IL-2R, IL-1β, IL-4, IL-8 and IL-17 are potential risk factors for severity and high mortality in COVID-19. Simultaneously, the upregulation of these cytokines may be driving factors for the sex and region predisposition.

## Introduction

Coronavirus disease 2019 (COVID-2019), caused by severe acute respiratory syndrome coronavirus 2 (SARS-CoV-2), has raised major public health crises since 2019. Though many patients with COVID-19 present no symptoms or only mild symptoms (including fever, cough, and fatigue), some suffer severe symptoms and may progress to pneumonia, acute respiratory distress syndrome (ARDS), multi organ dysfunction and even death. The severity of COVID-19 is known to be closely correlated to cytokines storms, when the immune system is unable to counteract the virus, cytokine storms in patients may lead to macrophage hyperactivity and further systemic abnormal reactions (Lang et al., 2020; Liang et al., 2020; Makaronidis et al., 2020). However, the characteristics of the cytokine storms in COVID-19 patients have not been fully illustrated.

In death cases, patients with COVID-19 shows a higher risk of mortality in males sex (Griffith et al., 2020). According to the largest sex-disaggregated data from 47 countries, men with COVID-19 have higher morbidity than women with COVID-19 (63.8% men; 36.2% women). In addition, the overall mortality of COVID-19 is more than 2.3 times higher in men than in women (Control and Response, 2020). The discrepancy in COVID-19 outcomes between male and female patients may be attributed to several biological and social factors, especially cytokine storms (Griffith et al., 2020). Moreover, as the Covid-19-related literatures grows increasingly, the racial and ethnic disparities showed that the death and severity rate of Asians are lower than the other population (Tirupathi et al., 2020a; Mackey et al., 2021a).

To this end, we conducted a systematic review and meta-analysis to identify the key factors associated to COVID-19 severity and death, especially in terms of the sex and race bias detected in severe COVID-19 patients.

## Methods

### Search Strategy

We screened databases (Web of Science, Embase, the Cochrane Library, and PubMed) from December 2019 to June 2021. We also registered on the INPLASY (International Platform of Registered Systematic Review and Meta analysis Protocols platform). The number for our study is INPLASY2021120050. To search for more articles, we also screened related reference lists from relevant studies. The search terms included (“2019 novel coronavirus disease”) OR (“COVID19”) OR (“COVID-19 pandemic”) OR (“SARS-CoV-2 infection”) OR (“COVID-19 virus disease”) OR (“2019 novel coronavirus infection”) OR (“2019-nCoV infection”) OR (“coronavirus disease 2019”) OR (“coronavirus disease-19”) OR (”2019-nCoV disease”) OR (“COVID-19 virus infection”) OR (“cytokines”).

### Inclusion and Exclusion Criteria

All the included studies met the following criteria: 1) the types of studies considered for inclusion were prospective or retrospective cohort studies comparing mild groups and severe groups; 2) the circulating levels of cytokines were analyzed before treatment. The exclusion criteria were reviews, studies of interventions other than cytokines, *in vitro* studies and *in vivo* animal experiments. To further reduce the accidental error of our study, each analysis of cytokines should contain more than two studies. Only English studies were screened in our study. After screening and collecting the literature, two authors removed duplicate publications by Endnote and independently evaluated each study based on their title and abstract. The symptom criteria are listed as follows.

For the mild group, patients had respiratory symptoms (fever, cough, fatigue, anorexia, headache), without evidence of viral pneumonia or hypoxia.

For the severe group, patients had one or more of the following conditions: respiratory distress, respiratory rate ≥30 times/minute, oxygen saturation (SpO2) ≤93% at rest, arterial partial pressure of oxygen (PaO2)/Fraction of inspiration O2 (FiO2) in arterial blood ≤300 mmHg, >50% lung imaging progress in the short term within 24–48 h, respiratory failure and mechanical ventilation required, shock, combined with other organ failure, and transfer to the intensive care unit (8).

### Data Extraction and Quality Assessment

Two authors (Hu & Pan) collected data from the included studies, including the first author, study country, inclusion time, age, sex, sample sizes, mild group/severe group, survivors/non-survivors, study design, and outcomes. Another two authors assessed the quality of the studies using the Newcastle-Ottawa Scale (NOS) and scored points for each included study independently.

### Statistical Analysis

Review Manager 5.3 was used to perform all statistical analyses. The mean and standard deviation (SD) were used as measurements across articles. We calculated the sample mean and SD by the sample size and interquartile range (IQR) (Wan et al., 2014; Luo et al., 2018). The circulating levels of cytokines between different groups were collected from the selected articles and analyzed using a random-effects model when I^2^>50%. The standard Cochran’s Q test and I^2^ statistics were also used to identify heterogeneity from the included articles. Significant heterogeneity was determined when I^2^ value > 50% and *p*-value <0.05.

## Results

### Large Scale Data From Clinical Reports

**FIGURE 1:**
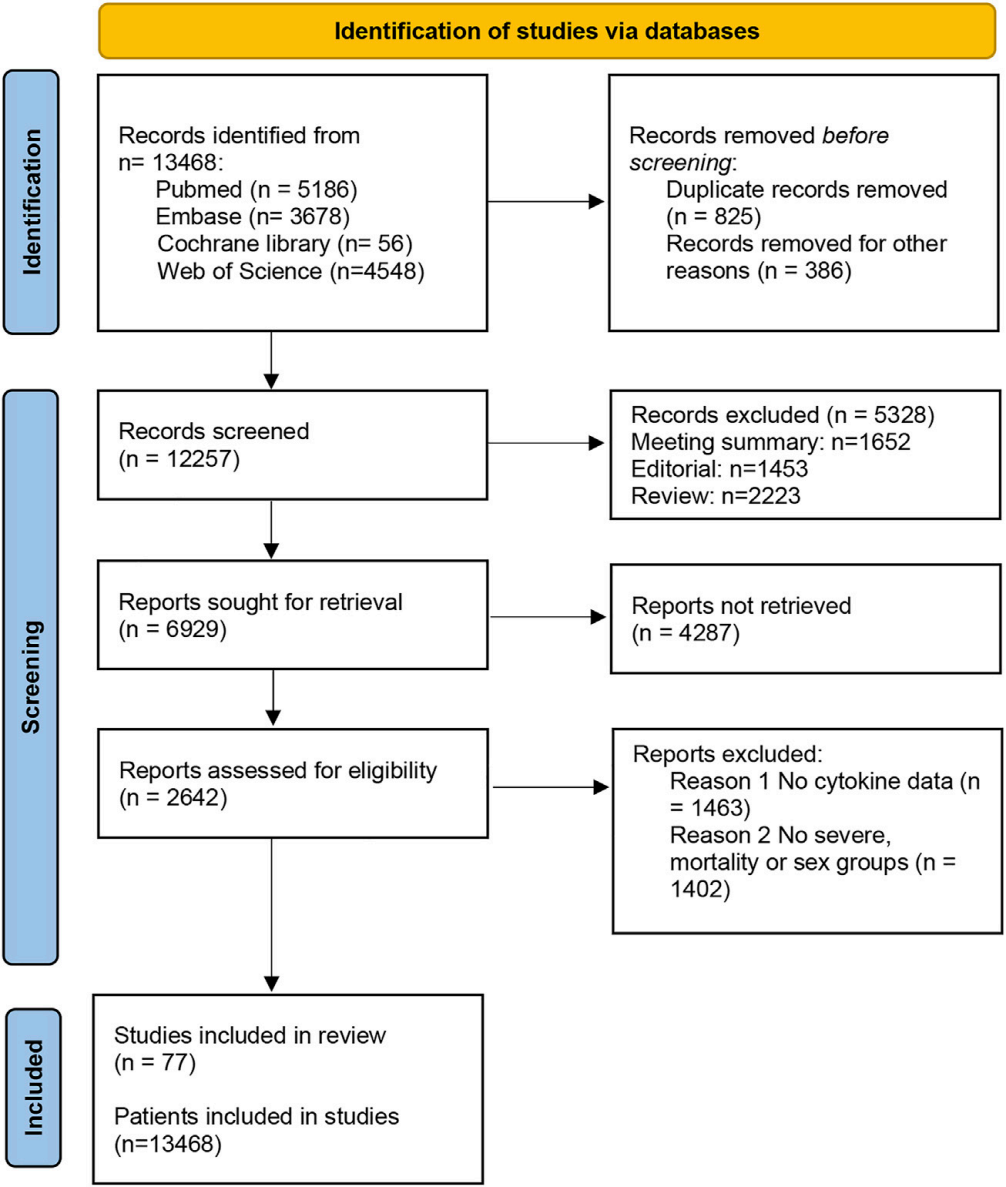
Literature search and screening process.

A total of 13,468 studies were screened out by the database search. After removing 826 duplicates, we excluded 8452 articles by reading the titles and abstracts of the studies. Then, we read the remaining literature and excluded studies that were not matched to the inclusion and exclusion criteria. There were 77 articles with 13,986 patients ultimately included in this study (Han et al., 2020; Yang et al., 2020; Rutkowska et al., 2021) (Figure 1). The baseline features of all included studies are presented in Table 1.

**TABLE 1:** Basic characteristics of 77 studies included in Meta-analysis.

Author	Study region	Inclusion time	Mean age (years)	gender	Sample sizes	Mild group/Severe groups or survival/non-survival groups	Study design	Outcomes	Journal types
Ai-Ping Yang Yang et al. (2020)	China	N/A	46.4	60% male	93	69/24	retrospective cohort	IL-6, IL-10, TNF-α, IL-1β, IL-4, IL-8, IL-17	Normal
Bo Xu Xu et al. (2020a)	China	26 Dec 2019 to 1 Mar2020	62	55% male	187	159/28	retrospective observational study	IL-6, IL-10, TNF-α, IL-1β	Normal
Changcheng Zheng Zheng et al. (2020a)	China	15 Feb 2020	60	43.6 male	55	34/21	retrospective observational study	IL-6	Normal
Changsong wang Wang et al. (2020a)	China	N/A	62.9	50% male	45	33/12	retrospective cohort	IL-6, IL-10, IL-4	Normal
Chaomin Wu Wu et al. (2020a)	China	25 Dec 2019, to 26 Jan 2020	51	43.7% male	201	117/84	retrospective cohort	IL-6	Normal
Chuan Qin Qin et al. (2020a)	China	Jan 10 to 12 Feb 2020	58	52% male	452	166/286	retrospective observational study	IL-6, IL-2R, IL-10, TNF-α, IL-8	Normal
Egon Burian Burian et al. (2020)	German	Mar and April 2020	61.54	35% male	65	37/28	retrospective cohort	IL-6	Normal
Fangfang Liu Liu et al. (2020a)	China	Jan 20 to 23 Feb 2020	48	55.38% male	65	42/23	retrospective cohort	IL-6	Normal
Fei Zhou Zhou et al. (2020a)	China	29 Dec 2019 to 31 Jan 2020	56	62% male	191	137/54	retrospective cohort	IL-6	Normal
Fengqin Zhang Zhang et al. (2020a)	China	Feb to March 2020	N/A	N/A	34	27/7	retrospective observational study	IL-6, IL-10, TNF-α, IL-8	Normal
Guang Chen Chen et al. (2020a)	China	Jan 2–7, 2020	56	81% male	21	10/11	retrospective observational study	IL-6, IL-2R, IL-10, TNF-α, IL-8	Normal
Haijun Wang (Wang et al., et al.)	China	Jan 2 to 5 Feb 2020	49	43.6% male	83	33/50	retrospective cohort	IL-6	Normal
Han Huang Han et al. (2020)	China	Jan 2020 and February 2020	N/A	50% male	102	42/60	retrospective cohort	IL-6, IL-10, TNF-α, IL-4	Normal
Hong Huang Huang et al. (2020)	China	Feb and March 2020	36	46% male	31	27/4	retrospective cohort	IL-6, IL-10, TNF-α, IL-2R	Normal
Hua Fan Fan et al. (2020)	China	30 Dec 2019 to 16 Feb 2020	58.36	67% male	73	47/26	retrospective observational study	IL-6	Normal
Huizheng Zhang Zhang et al. (2020b)	China	N/A	N/A	51.2% male	43	29/14	retrospective observational study	IL-6, IL-10, TNF-α, IL-17	Preprint
Jia Ma Ma et al. (2020)	China	1 Jan 2020 to 30 Mar 2020	62	54.5% male	37	17/20	retrospective observational study	IL-6	Normal
Lang Wang Wang et al. (2020b)	China	Jan 1 to 6 Feb 2020	71	49% male	339	274/65	retrospective observational study	IL-6	Normal
Lei Liu Liu et al. (2020b)	China	N/A	45	62.7% male	51	44/7	retrospective case series	IL-6	Preprint
Lucas Quartuccio Quartuccio et al. (2020)	Italy	N/A	66.5	79% male	24	18/6	retrospective cohort	IL-6	Normal
Maria effenberger Effenberger et al. (2020)	Austria	26th February to 21st April 2020	60.69	62.5% male	96	81/15	retrospective case series	IL-6	Normal
María J. Pérez-Sáez Pérez-Sáez et al. (2020)	Spain	18th March 2020	59.3	67.5% male	80	54/26	retrospective case series	IL-6	Normal
Mario Fernández‐Ruiz Fernández-Ruiz et al. (2020)	Spain	16th March to 27th March 2020	46.8	65.9% male	88	39/49	retrospective cohort	IL-6	Normal
Marta Crespo Crespo et al. (2020)	Spain	Mar to April 2020	71	75% male	16	8/8	Prospective cohort study	IL-6	Normal
Miao Luo Luo et al. (2020)	China	Jan and March 2020	61	51.2% male	1018	817/201	retrospective cohort	IL-2R, IL-6, IL-10, TNF-α, IL-8	Normal
Michael Dreher Dreher et al. (2020)	German	Feb and March 2020	65	66% male	50	26/24	retrospective case series	IL-6	Normal
Ming Ni Ni et al. (2020)	China	1 to 21 February 2020	60	50% male	27	male 14/female 13	retrospective case series	IL-6, IL-10, TNF-α	Normal
Paola Toniati Toniati et al. (2020)	Italy	Mar 9th and 20 Mar 2020	62	88% male	100	77/23	retrospective case series	IL-6	Normal
Pingzheng Mo Mo et al. (2020)	China	Jan 1st to 5 Feb 2020	54	55.5 male	155	70/85	retrospective observational study	IL-6	Normal
Qin Lu Qin et al. (2020b)	China	26 January 2020 and 5 February 2020	55.2	57.9 male	233	135/98	retrospective cohort	IL-6, IL-2R, IL-10, TNF-α	Normal
Qiurong Ruan Ruan et al. (2020)	China	N/A	N/A	N/A	150	82/68	retrospective observational study	IL-6	Normal
Ruirui Wang Wang et al. (2020c)	China	Jan 20 to 9 Feb 2020	38.7	57% male	125	100/25	retrospective descriptive study	IL-6	Normal
Shaohua Li Li et al. (2020a)	China	20 Jan 2020, to 20 Mar 2020	48.5	58% male	69	43/26	retrospective cohort	IL-6, TNF-α, IL-1β, IL-8	Normal
Susu He He et al. (2020)	China	Jan 17 to 12 Feb 2020	44.5	53% male	93	60/33	retrospective cohort	IL-6. IL-10	Normal
Suxin Wan Wan et al. (2020)	China	26 January to 4 February 2020	43.1	53.6% male	123	102/21	retrospective observational study	IL-6, IL-10, TNF-α, IL-4, IL-17	Normal
Takahisa Mikami Mikami et al. (2020)	United States	Mar and April 2020	59	54.5% male	2820	2014/806	retrospective cohort	IL-6, TNF-α, IL-8	Normal
Tao Chen Chen et al. (2020b)	China	13 January to 12 February 2020	62	62% male	274	161/113	retrospective descriptive study	IL-6 IL-2R, IL-10 TNF-α, IL-8	Normal
TAO Liu Liu et al. (2020c)	China	December 2019 to July 2020	53.9	42.2% male	77	11/66	retrospective cohort	IL-6, IL-10	Normal
Tielong Chen Chen et al. (2020c)	China	1 Jan 2020, to 10 Feb 2020	54	53.2% male	55	36/19	retrospective case series	IL-6	Normal
Tobias Herold Herold et al. (2020)	German	Feb 29 to 27 Mar 2020	61	70% male	89	57/32	retrospective case series	IL-6	Normal
Wenjun Tu Tu et al. (2020)	China	3 Jan to 24 February 2020	70	76% male	174	149/25	retrospective case series	IL-6	Normal
Xiaohong Yuan Yuan et al. (2020)	China	Feb 15 to 30 Mar 2020	67	47.9% male	117	61/56	retrospective cohort	IL6, IL-10, IL-4	Normal
Xia Xu Xu et al. (2020b)	China	3 Feb 2020, to 20 Mar 2020	57	40.91% male	88	47/41	retrospective descriptive study	IL-6 IL-2R, TNF-α, IL-8	Normal
Xiong Bei (Xiong et al., 2020)	China	21 Mar 2020	66	61.4% male	57	19/38	retrospective case series	IL-6	Normal
Yang Liu Liu et al. (2020d)	China	22 Jan 2020, to 15 Feb 2020	45	64.4% male	76	46/30	retrospective case series	IL-6, IL-2R, IL-10, IL-1β, IL-8	Normal
Yang Xu Xu et al. (2020c)	China	N/A	57	50.7% male	69	44/25	retrospective cohort	IL-6	Preprint
Yang Xu 2 Xu (2020)	China	N/A	N/A	N/A	10	8/2	retrospective observational study	IL-6	Preprint
Yang Zhao Zhao et al. (2020)	China	Jan 13 and 4 Mar 2020	58	47.3% male	539	414/125	retrospective observational study	IL-6	Normal
Yangjing Xie Xie et al. (2020)	China	Feb and March 2020	66	43.5% male	62	38/24	retrospective cohort	IL-6	Normal
Yanli Wang Wang et al. (2020d)	China	25 Jan 2020 and 8 Mar 2020	52	65% male	43	35/8	retrospective observational study	IL-6, IL-10, IL-4	Normal
Yaqing Zhou Zhou et al. (2020b)	China	28 Jan 2020 to 2 Mar 2020	66	65.9% male	21	8/13	retrospective case series	IL-6	Normal
Yi Li Li et al. (2020b)	China	28 January 2020, to 12 March 2020	6	56.8% male	125	48/77	retrospective case series	IL-6, IL-10, TNF-α, IL-4	Normal
Ying Chi Chi et al. (2020)	China	N/A	45.21	56% male	66	58/8	retrospective case series	IL-6, IL-2R, IL-10, TNF-α, IL-1β, IL-4, IL-8, IL-17	Normal
Yingjie Wu Wu et al. (2020b)	China	29 December 2019 to 20 February 2020	61	63.3% male	71	32/39	retrospective case series	IL-6, IL-10, TNF-α, IL-4	Normal
Ying Sun Sun et al. (2020)	China	N/A	47	58.7% male	63	19/44	retrospective case series	IL-6	Normal
Yi Zheng Zheng et al. (2020b)	China	Jan. 22 and Mar. 5, 2020	66	67.6% male	34	19/15	retrospective cohort	IL-6, IL-10	Normal
Yong Gao Gao et al. (2020)	China	23 Jan 2020 to 2 Feb 2020	44	60.6% male	43	28/15	retrospective case series	IL-6	Normal
Zhe Zhu Zhu et al. (2020)	China	Jan 23 to Feb20, 2020	50.9	36.43% male	127	111/16	retrospective cohort	IL-6, IL-10, TNF-α, IL-4	Normal
Zhihua Lv Lv et al. (2020)	China	4 Feb 2020 to Feb28, 2020	62	49.4% male	354	115/239	retrospective cohort	IL-6, IL-10, TNF-α, IL-4	Normal
Zhilin Zeng Zeng et al. (2020)	China	28 Jan 2020, to 12 Feb 2020	62	51.1% male	317	93/224	retrospective cohort	IL-6, IL-2R, IL-10, TNF-α	Normal
Zhongliang Wang Wang et al. (2020e)	China	Dec 2019 to February 2020	42	46% male	69	55/14	retrospective cohort	IL-6, IL-10, TNF-α, IL-4	Normal
Sophie Hue Hue et al. (2020)	France	Mar 2020	60	91% male	38	25/13	retrospective cohort	IL-6, IL-10	Normal
Elzbieta Kalicinska Kalicińska et al. (2021)	Poland	Dec 2020	62	52% male	82	51/31, 54/28	Prospective cohort	IL-6, TNF-α	Normal
Dianming Li Li et al. (2020c)	China	Mar 2020	56	62.5% male	65	41/24	retrospective cohort	IL-6	Normal
Francisco Javier Gil-Etayo Gil-Etayo et al. (2021)	Spain	Sep 2020	55	67% male	34	28/6	Prospective cohort	IL-6, IL-10	Normal
Feng Gao Gao et al. (2021)	China	Feb 2020	49	42.5% male	121	102/19	retrospective cohort	IL-6, IL-10	Normal
Wei Zhu Zhu et al. (2021)	China	Mar 2020	65	45% male	1106	675/431	retrospective cohort	IL-6, IL2R, TNF-α, IL-8	Normal
Zirui Meng (Meng et al. (2021)	China	Apr 2020	48	53% male	98	71/27	retrospective cohort	IL-6, IL-10, TNF-α, IL-8	Normal
Chenze Li Li et al. (2020d)	China	Apr 2020	63	49.6% male	989	770/219, 141/78	retrospective cohort	IL-6, IL-2R, IL-10, TNF-α, IL-8	Normal
Brahim Belaid Belaid et al. (2021)	Algeria	Apr 2020	59	70.18% male	57	31/26	retrospective cohort	IL-6, TNF-α	Normal
Rocio Laguna-Goya Laguna-Goya et al. (2020)	Spain	Apr 2020	52	63.3% male	501	465/36	Prospective cohort	IL-6	Normal
Jose J. Guirao Guirao et al. (2020)	Spain	Apr 2020	65	52% male	50	42/8, 36/14	retrospective cohort	IL-6	Normal
Jose Marıa Galvan-Roman Galván-Román et al. (2021)	Spain	Mar 2020	63	66% male	146	102/44	retrospective cohort	IL-6	Normal
Li-Da Chen (hen et al. (2020d)	China	Mar 2020	52	50% male	94	69/25	retrospective cohort	IL-6, IL-2R, TNF-α, IL-8	Normal
Lucía Guillén Guillén et al. (2020)	Spain	Apr 2020	62	73% male	64	49/15	retrospective cohort	IL-6	Normal
Enrico Maria Trecarichi Trecarichi et al. (2020)	Italy	May 2020	80	57.1% male	48	34/14	retrospective cohort	IL-6	Normal
Elzbieta Rutkowska Rutkowska et al. (2021)	Poland	Jan 2021	56	56% male	38	23/15	retrospective cohort	IL-6	Normal

Studies were published between December 2019 and June 2021. Among the 77 studies, 57 studies were performed in China, eight in Spain, three in Germany, three in Italy, two in Poland, and one each in Austria, the USA, France and Algeria. Seventy-three studies were published in normal journals, and four were published in preprint journals.14 cytokines were reported in these 77 studies, including IL-1β, IL-2, IL-2R, IL-4, IL-5, IL-6, IL-8, IL10, IL-15, IL-17, TNF-α, IFN-γ, MCP-1, and CXCL-10. Review Manager 5.3 was used to calculate and compare the sample mean and SD by the sample size and interquartile range. After removing the cytokines that having no statistical difference in either severe or death group, the cytokines that only contain two articles were also removed. Totally eight cytokines were included in our meta-analysis, containing IL-1β, IL-2R, IL-4, IL-6, IL-8, IL-10, IL-17, and TNF- α. Furthermore, we also screened the cytokines associated with gender or regions of COVID-19 patients. IL-2R, IL-6, IL-10 and TNF-α, which were correlated with gender or regions of COVID-19 patients, were finally presented in this study. All the included studies detected the serum levels of IL-6, while 13 studies focused on IL-2R, 31 studies analyzed IL-10 and 29 studies were related to the serum levels of TNF-α. Five studies analyzed IL-1β, 12 studies analyzed IL-4, 11 studies analyzed IL-8 and IL-17 was studies by four studies. Moreover, five studies analyzed the correlation between genders and cytokines. Fifty-seven and twenty-four studies analyzed the serum levels of cytokines in severity and mortality groups. All 77 studies had NOS quality scores greater than 6, indicating that all these studies have high levels of quality, as shown in Table 2.

**TABLE 2:** Methodological quality of the 77 studies based on the NOS for studies.

First author	Study design	Selection	Comparability	Assessment of outcome	Total quality scores
Ai-Ping Yang	Cohort	***	**	**	7
Bo Xu	Cohort	***	**	***	8
Changcheng Zheng	Cohort	**	**	***	7
Changsong wang	Cohort	***	**	***	8
Chaomin Wu	Cohort	***	**	**	7
Chuan Qin	Cohort	****	**	***	9
Egon Burian	Cohort	***	**	**	7
Fangfang Liu	Cohort	***	**	**	7
Fei Zhou	Cohort	**	**	***	7
Fengqin Zhang	Cohort	***	**	***	8
Guang Chen	Cohort	***	**	**	7
Haijun Wang	Cohort	***	**	**	7
Han Huang	Cohort	***	**	***	8
Hong Huang	Cohort	****	**	***	9
Hua Fang	Cohort	***	**	**	7
Huizheng Zhang	Cohort	***	**	***	8
Jia Ma	Cohort	***	**	**	7
Lang Wang	Cohort	****	**	***	9
Lei Liu	Cohort	****	**	***	9
Lucas Quartuccio	Cohort	***	**	**	7
Maria effenberger	Cohort	***	**	***	8
María J. Pérez-Sáez	Cohort	***	**	**	7
Mario Fernández‐Ruiz	Cohort	****	**	***	9
Marta Crespo	Cohort	****	**	*	7
Miao Luo	Cohort	***	**	***	8
Michael Dreher	Cohort	***	**	***	8
Ming Ni	Cohort	***	**	***	8
Paola Toniati	Cohort	***	**	**	7
Pingzheng Mo	Cohort	****	**	*	7
Qin Lu	Cohort	****	**	***	9
Qiurong Ruan	Cohort	****	**	*	7
Ruirui Wang	Cohort	**	**	***	7
Shaohua Li	Cohort	**	**	***	7
Sophie Hue	Cohort	***	**	***	8
susu He	Cohort	****	**	*	7
Suxin Wan	Cohort	***	**	***	8
Takahisa Mikami	Cohort	****	**	**	8
Tao Chen	Cohort	***	**	***	8
TAO Liu	Cohort	***	**	***	8
Tielong Chen	Cohort	****	**	*	7
Tobias Herold	Cohort	****	**	***	9
Wenjun Tu	Cohort	***	**	***	8
Xia Xu	Cohort	****	**	**	8
Xiaohong Yuan	Cohort	***	**	***	8
Xiong Bei	Cohort	***	**	***	8
Yang Liu	Cohort	**	**	***	7
Yang Xu	Cohort	**	**	***	7
Yang Xu 2	Cohort	****	**	***	9
Yang Zhao	Cohort	***	**	***	8
Yangjing Xie	Cohort	***	**	***	8
Yanli Wang	Cohort	***	**	***	8
Yaqing Zhou	Cohort	****	**	**	8
Yi Li	Cohort	***	**	***	8
Yi Zheng	Cohort	***	**	**	7
Ying Chi	Cohort	***	**	***	8
Ying Sun	Cohort	***	**	***	8
Yingjie Wu	Cohort	****	**	***	9
Yong Gao	Cohort	***	**	***	8
Zhe Zhu	Cohort	**	**	**	6
Zhihua Lv	Cohort	****	**	***	9
Zhilin Zeng	Cohort	***	**	***	8
Zhongliang Wang	Cohort	***	**	***	8
Elzbieta Kalicinska	Cohort	****	**	***	9
Dianming Li	Cohort	***	**	***	8
Francisco Javier Gil-Etayo	Cohort	***	**	***	8
Feng Gao	Cohort	***	**	***	8
Wei Zhu	Cohort	**	**	***	7
Zirui Meng	Cohort	***	**	***	8
Chenze Li	Cohort	****	**	***	9
Brahim Belaid	Cohort	****	**	**	8
Rocio Laguna-Goya	Cohort	****	**	***	9
Jose J. Guirao	Cohort	***	**	***	8
Jose Marıa Galvan-Roman	Cohort	****	**	***	9
Li-Da Chen	Cohort	****	**	*	7
Lucía Guillén	Cohort	***	**	**	7
Enrico Maria Trecarichi	Cohort	***	**	***	8
Elzbieta Rutkowska	Cohort	***	**	***	8

### Proinflammatory Cytokines as the Driving Factor for Severity and High Mortality in COVID-19 Patients

To determine whether the circulating levels of inflammatory cytokines are risk factors for severity and mortality of COVID-19 patients, we classified the patients into mild and severe groups. There were 57 studies and 7,807 patients included in this meta-analysis. Compared to patients in the mild group, circulating levels of IL-6 was found to be significantly increased in patients in the severe group (19.76 [16.59, 22.93], *p* < 0.00001, Supplementary Figure S1). The serum level of IL-6 in the non-surviving group was also significantly elevated compared with that in the surviving group (52.33 [44.16, 60.50], *p* < 0.00001, Supplementary Figure S2). In addition to IL-6, the serum levels of IL-2R, IL-10, IL-1β, IL-4, IL-8, IL-17 and TNF-α were also elevated in both severe and non-surviving COVID-19 patients (Supplementary Figures S3–S6). Suggesting that the upregulation of these cytokines were correlated with the prognosis of COVID-19 patients.

### Alterations of the Distinctive Cytokines Are Related to Sex Bias in COVID-19 Patients

**FIGURE 2:**
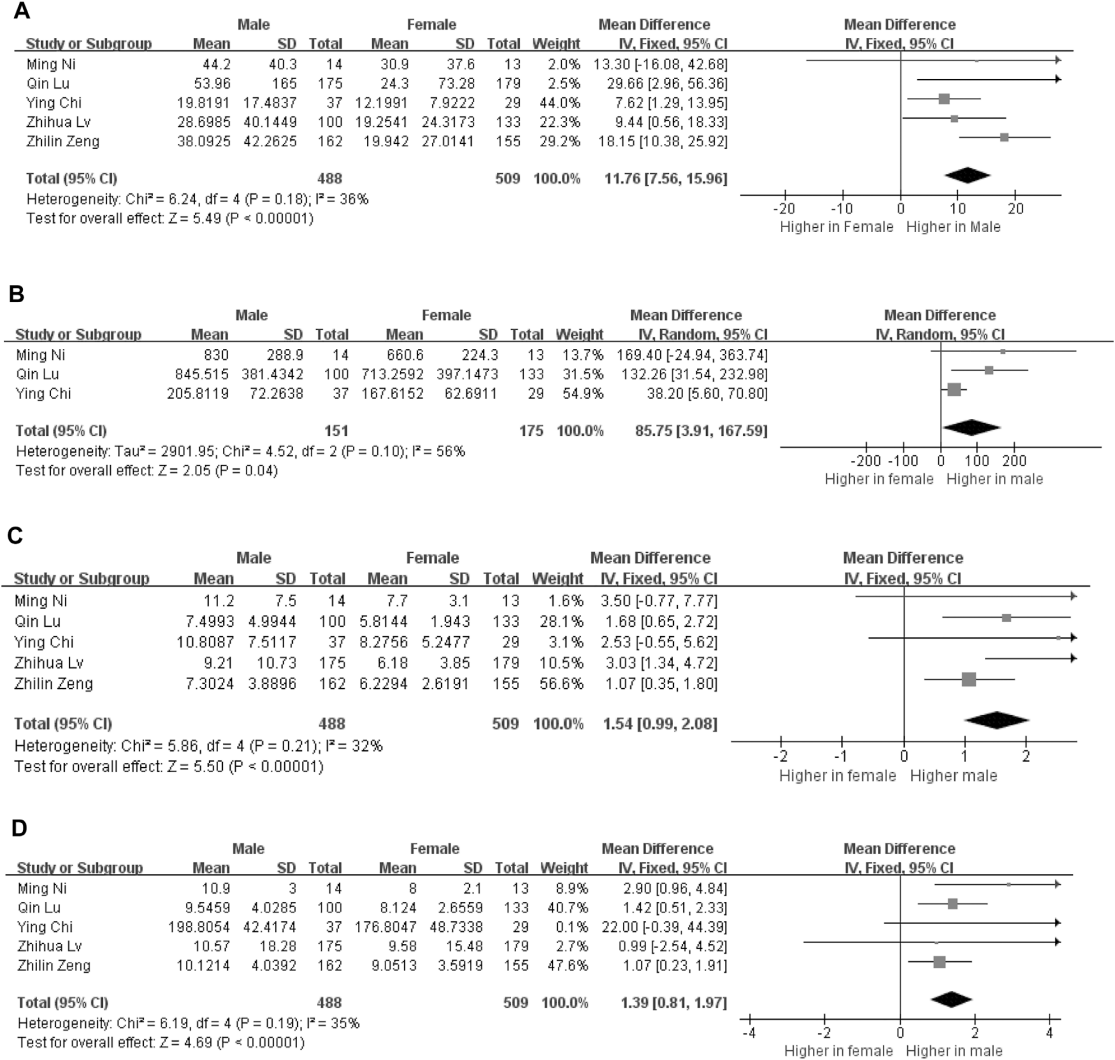
Forest plot for the male and female groups. The serum levels of IL-6 levels in the groups of male and female **(A)**. The serum levels of IL-2R levels in the groups of male and female **(B)**. The serum levels of IL-10 levels in the groups of male and female **(C)**. The serum levels of TNF-α levels in the groups of male and female **(D)**.

In this meta-analysis, four cytokines were found to be correlated with severity of male COVID-19 patients. Five studies reporting circulating interleukin-6 (IL-6) levels in male (*n* = 488) and female (*n* = 509) COVID-19 patients were included. In addition, interleukin-2 receptor (IL-2R), interleukin-10 (IL-10) and tumor necrosis factor α (TNF-α) were also different between male and female patients. Compared to female patients, the expression levels of circulating IL-6 (11.76 [7.56, 15.96], *p* < 0.000001), IL-2R (85.75 [3.91, 167.59], *p* = 0.04), IL-10 (1.54 [0.99, 2.08], *p* < 0.00001) and TNF-α (1.39 [0.81, 1.97], *p* < 0.00001) were found to be significantly elevated in male patients (Figure 2). Additionally, we conducted a sensitivity analysis to confirm the robustness of the model, and a significant sex gap was detected in circulating levels of IL-6, IL-2R, IL-10 and TNF-α.

### The Levels of IL-6 Related to Severity and High Mortality in COVID-19 Patients From Different Continents

**FIGURE 3:**
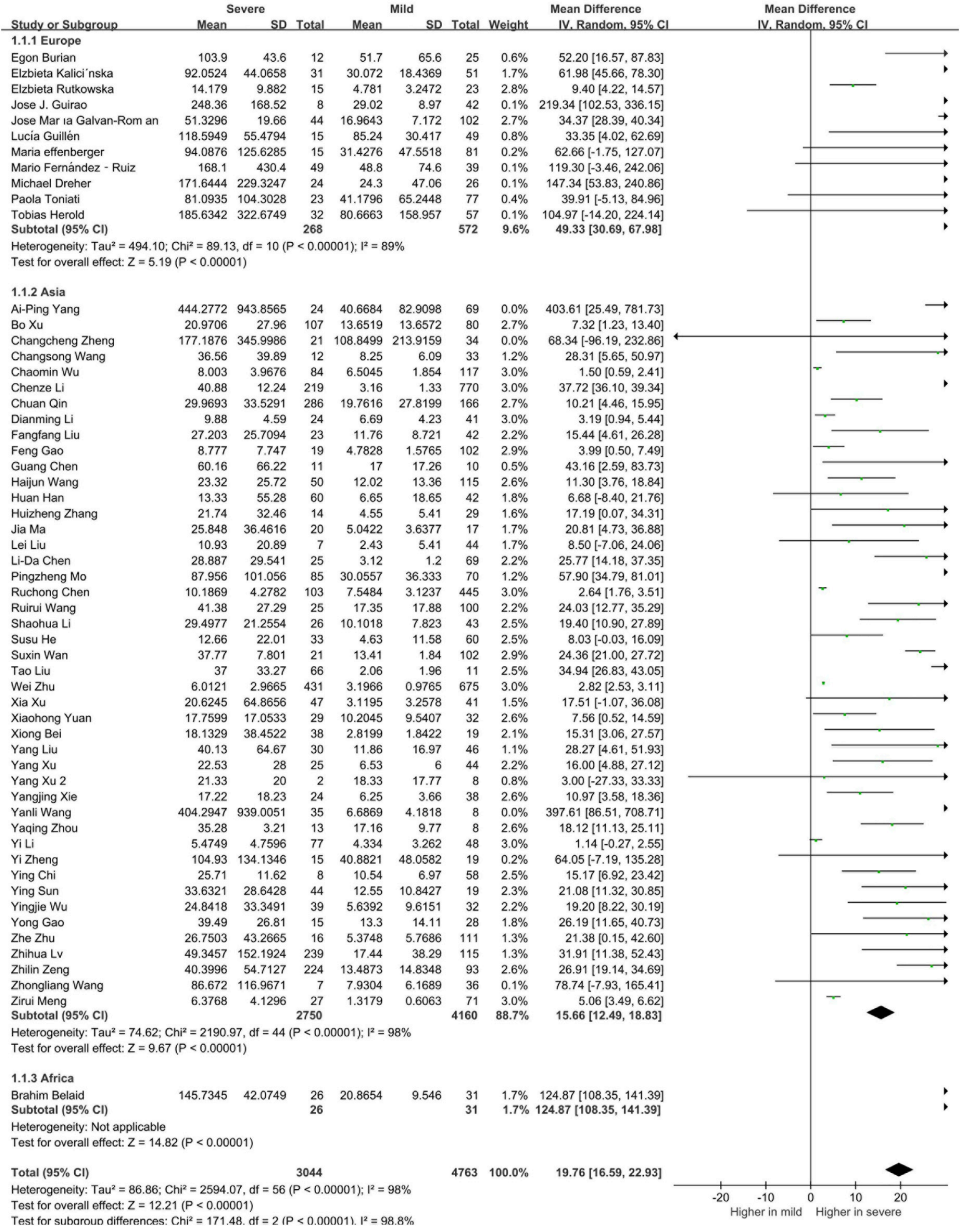
The serum levels of IL-6 in the different continent of mild and severe.

**FIGURE 4:**
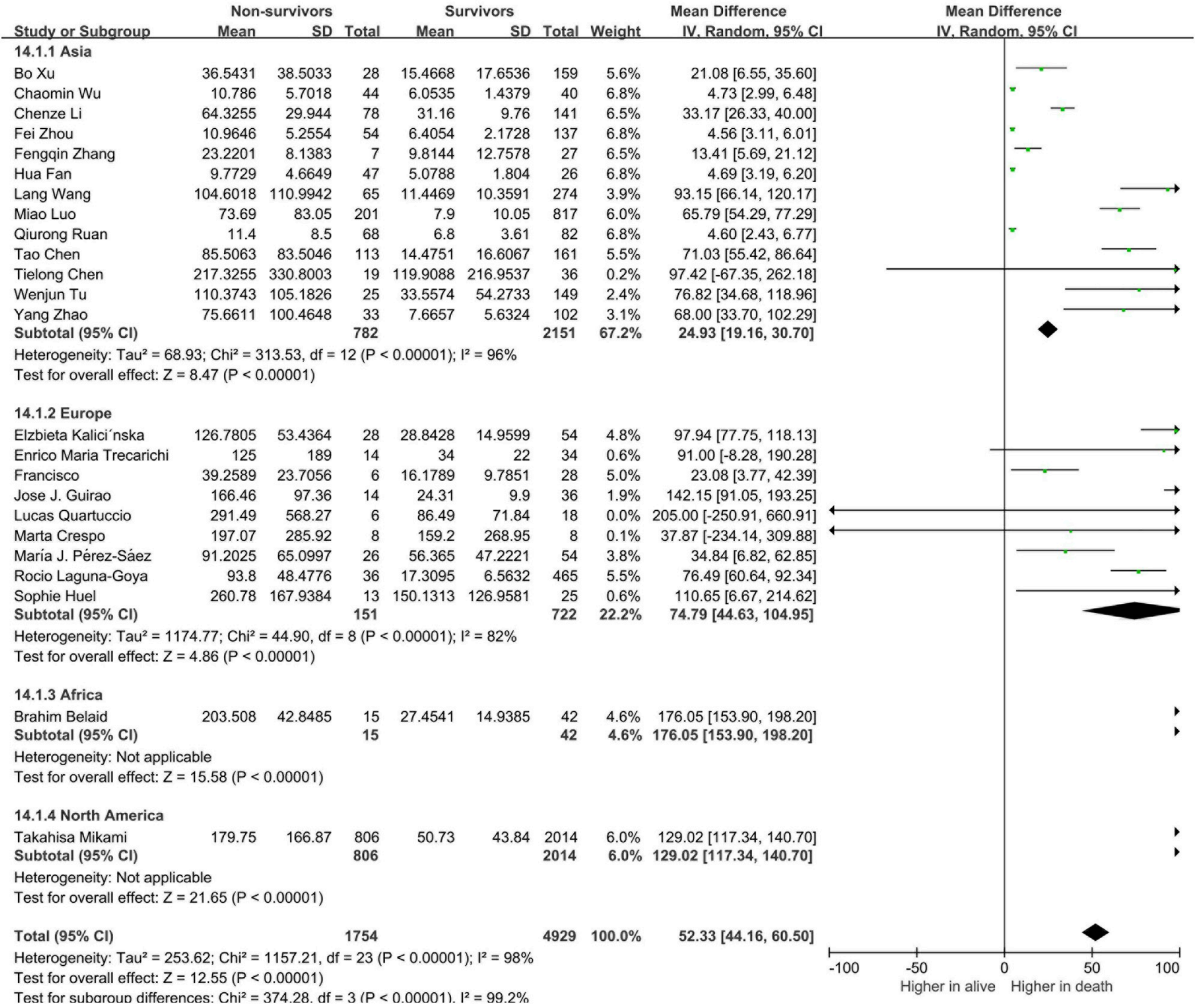
The serum levels of IL-6 in the different continents of the alive and death.

We further analyzed the correlation between cytokines and continents. We classified the articles into Asia, Europe, Africa and North America groups, and there were 840 European patients, 6,910 Asian patients and 57 African patients in the selected studies. To better interpret the differences between countries, we compared the ages, sex distributions and the severe rate of the included patients in the two territories. Results showed that ages and the proportions of severe or dead patients were comparable, while the male patients in the severe COVID-19 patients in Europe was significantly higher than that in Asia (Supplementary Tables 1, 2). The results of our meta-analysis showed that Asian, European, and African patients with severe COVID-19 had elevated circulating IL-6 levels and the circulating IL-6 levels of European and African was higher than the Asian patients (Figure 3). Notably, we found that there were 997 Asian, 223 European, 19 African and 1007 North American in the analysis of mortality. Among them, all the death patients with COVID-19 had higher IL-6 levels than the survive patients. Moreover, Asian death patients still the have the lowest circulating IL-6 levels than the other continents’ patients (Figure 4). Unlike IL-6, the serum level of IL-10 had the potential to predict the risk of mortality in Asian patients, but it showed no correlation with mortality in European patients (Supplementary Figure S7).

## Discussion

**FIGURE 5:**
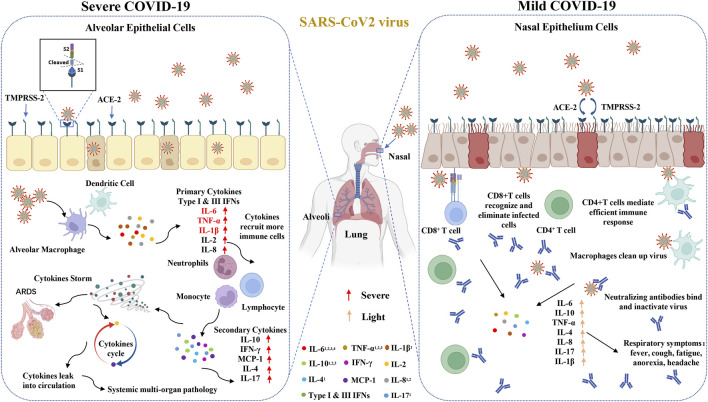
Increased circulating cytokines affect the development of COVID-19. The SARS-CoV-2 S protein engages with the host ACE2 receptor and is subsequently cleaved at S1/S2 and S2’ sites by TMPRSS2 protease. In the severe patients, COVID-19 invades the alveoli and activates innate immune responses to primary cytokines, such as type I and III IFNs, IL-6 and TNF-α, which further evokes the secondary cytokines and leads to cytokines storm. In the mild patients, immune cells have the ability of eliminating viruses and inhibiting them from invading alveoli, which leads to down-regulate cytokines in serum. ^1^The cytokines have significant differences between mild and severe groups. ^2^The cytokines between alive and death groups have significant differences. ^3^This cytokines have significant differences between male and female groups. ^4^This cytokines have significant differences between different regions.

The SARS-CoV-2 S protein engages with the host ACE2 receptor and is subsequently cleaved at S1/S2 and S2′ sites by TMPRSS2 protease, which leads to activation of the S2 domain and drives fusion of the viral and host membranes. The secretion of interferon is the first step to start the antiviral program. Alveolar cells are an important part of the epithelial endothelial barrier. After respiratory epithelial cells were first infected by virus, virus infection activates pattern recognition receptors in these cells, triggering the production and release of type I and type III interferons (IFNs) and other proinflammatory mediators (such as cytokines, chemokines and antimicrobial peptides), so as to start the host’s innate and acquired immune response, which further activated the secondary cytokines (such as IL-10, IFN-γ, MCP-1, IL-4, and IL-17) and lead to cytokines storm (Vabret et al., 2020). In the mild patients, immune cells have the ability of eliminating viruses completely and inhibit the them from invading alveoli, which lead to low cytokines in serum (Figure 5). In this study, we identified that the serum levels of IL-6, IL-2R, IL-10, TNF-α, IL-1β, IL-4, IL-8 and IL-17 were significantly elevated in the severe or death cases and probably play crucial roles in the progression of COVID-19. Male sex was identified as a hazard for more severe disease and higher mortality in COVID-19 (Takahashi et al., 2020; Zeng et al., 2020). The recognition of how sex influences COVID-19 outcomes have important significance for clinical management and remission tactics. In this large-scale worldwide meta-analysis, the related cytokines affecting the development of severe disease in male patients were identified and the serum of IL-6, as well as IL-10, IL-2R and TNF-α, in males was obviously higher than that in females.

IL-6, the core factor of “cytokine storm”, plays a pivotal role in the severity and high mortality of COVID-19. It enhances the production of TNF-α and IL-8 by stimulating the differentiation of T follicular helper cells, inhibits antiviral helper T cell 1 (Th1) cell commitment and improves the differentiation of helper T cell 2 (Th2) cells by regulating the circulating of IL-4 and interferon γ (IFN-γ) (Ahmadpoor and Rostaing, 2020; Wu and Yang, 2020). Moreover, elevated levels of IL-6 lead to acute lung injury and suppress the functions of T lymphocytes, macrophages and dendritic cells, which impair the immune system (Zhang et al., 2004). Tocilizumab, an IL-6 antagonist, revealed good capacity in inhibiting inflammation and cytokine storms in COVID-19 and various clinical studies have verified the beneficial effect of IL-6 and its receptor antagonists in treating severe and critical COVID-19 patients (Xu et al., 2020d; Potere et al., 2021). Besides IL-6, TNF-α inhibitor can also reduce lung exudation and inflammatory reactions, it has been used in the treatment of patients with covid-19 patients (Tirupathi et al., 2020b; Mackey et al., 2021b). However, blocking IL-6 and TNF-α inhibitor may not be used to all patients due to its potential adverse events and expensive price (Wang et al., 2020f; Keewan et al., 2021). The identification of which COVID-19 patients are suitable for treatment with IL-6 antagonists and TNF-α inhibitor are meaningful in the clinic. In our study, the cytokines IL-6, IL-10, and TNF-α were significantly upregulated in severe COVID-19 patients, especially in male patients, indicating that IL-6 antagonists and TNF-α inhibitors are more appropriate used in male patients to reduce both severity and mortality rate of COVID-19.

An increasing number of studies have pointed out that there are ethnicity-related differences in cytokines in systemic lupus erythematosus, chronic rhinosinusitis and other autoimmunity diseases (Niewold et al., 2012; Wang et al., 2016; Slight-Webb et al., 2020). We also focused on ethnicity-related differences in cytokines in COVID-19 patients and the results showed that there were lower circulating levels of IL-6 in Asian patients than in European and African patients, suggesting that IL-6 antagonists are recommended to use earlier in western countries.

This study had some limitations. Firstly, the articles that described the differential serum levels of cytokines in males and females were all from China. More clinical experiments should focus on the sex bias of cytokines in COVID-19. Secondly, our meta-analysis mainly investigated studies written in English, which might lead to language bias.

## Conclusion

These large-scale data revealed that the serum levels of IL-6, IL-10, IL-2R, TNF-α, IL-1β, IL-4, IL-8, and IL-17 are potential risk factors for severity and high mortality in COVID-19. The IL-6 antagonist and TNF-α inhibitor are likely to be a proper therapeutic strategy to reduce mortality in males with COVID-19 and in Western countries.
