# Supplementary material for: Increased Circulating Cytokines Have a Role in COVID-19 Severity and Death With a More Pronounced Effect in Males: A Systematic Review and Meta-Analysis
Source: Front Pharmacol. 2022 Feb 14;13:802228. doi: 10.3389/fphar.2022.802228 (PMC8883392; doi:10.3389/fphar.2022.802228)
Supplement: Supplementary file 1 [file Image5.PDF]

A

Study or Subgroup	Non-survivors Mean	SD	Total	Survivors Mean	SD	Total	Weight	Mean Difference IV, Random, 95% CI
Chenze Li	1,101.98	183.9	75	893.27	161.09	133	29.4%	208.71 [158.89, 258.53]
Fengqin Zhang	1,729.5	830.821	7	558.114	289.202	27	13.3%	1171.39 [546.32, 1796.45]
Miao Luo	1,092.3481	564.7903	201	478.1682	280.8164	817	29.1%	614.18 [533.76, 694.60]
Tao Chen	1,295.925	660.8278	113	624.3891	306.9253	161	28.2%	671.54 [540.80, 802.28]
**Total (95% CI)**			**396**			**1138**	**100.0%**	**585.08 [278.08, 892.08]**

Heterogeneity: Tau² = 82670.50; Chi² = 102.08, df = 3 (P < 0.00001); I² = 97%
Test for overall effect: Z = 3.74 (P = 0.0002)

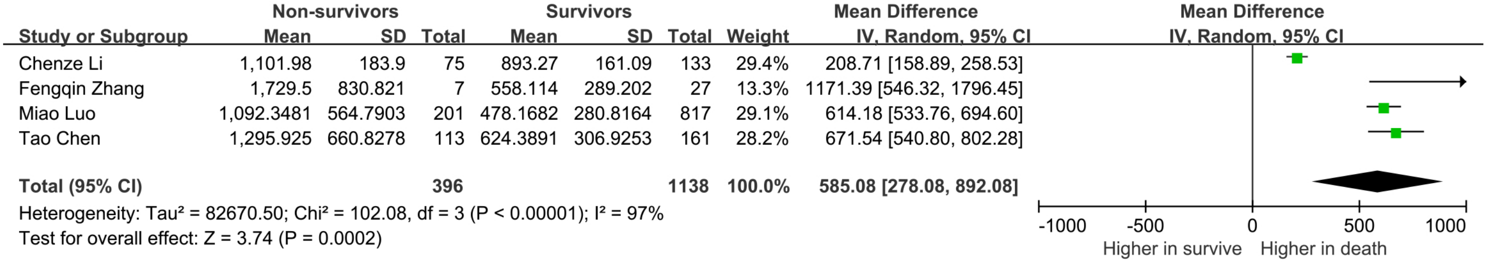

B

Study or Subgroup	Non-survivors Mean	SD	Total	Survivors Mean	SD	Total	Weight	Mean Difference IV, Random, 95% CI
Bo Xu	14.1585	13.2719	28	7.2841	4.645	159	10.7%	6.87 [1.91, 11.84]
Chenze Li	9.98	1.996	77	6.25	1.05	133	30.0%	3.73 [3.25, 4.21]
Fengqin Zhang	33.6116	41.5824	7	6.0359	2.2698	27	0.4%	27.58 [-3.24, 58.39]
Francisco	15.3109	10.4015	6	9.2648	3.748	28	4.8%	6.05 [-2.39, 14.48]
Miao Luo	10.36	7.62	201	5.43	0.74	817	28.1%	4.93 [3.88, 5.98]
Sophie Huel	524.708	88.8695	13	407.6953	148.3405	25	0.1%	117.01 [41.42, 192.61]
Tao Chen	13.7848	8.1102	113	6.1941	2.5434	161	25.9%	7.59 [6.04, 9.14]
**Total (95% CI)**			**445**			**1350**	**100.0%**	**5.70 [3.68, 7.72]**

Heterogeneity: Tau² = 3.48; Chi² = 36.03, df = 6 (P < 0.00001); I² = 83%
Test for overall effect: Z = 5.53 (P < 0.00001)

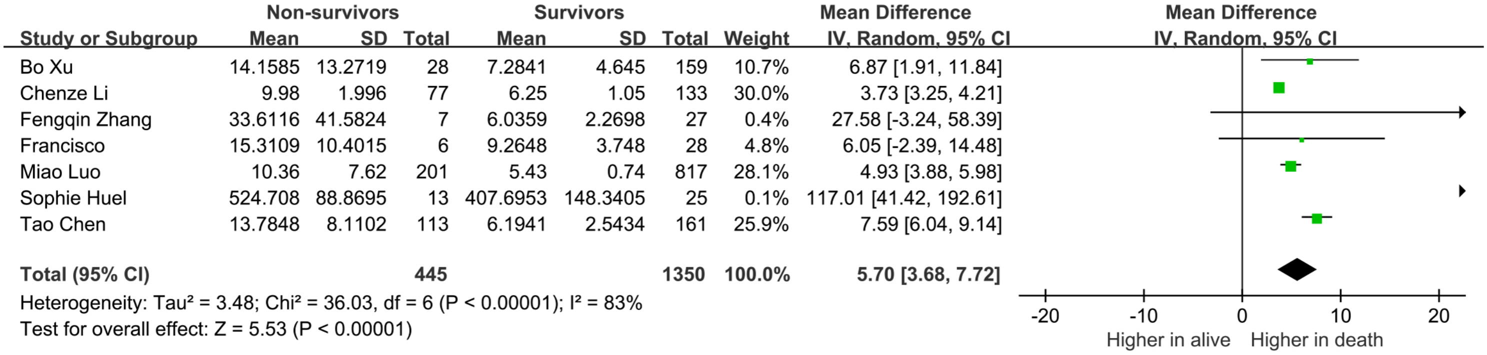

C

Study or Subgroup	Non-survivors Mean	SD	Total	Survivors Mean	SD	Total	Weight	Mean Difference IV, Random, 95% CI
Bo Xu	12.5213	3.5152	28	11.5238	5.6933	159	12.4%	1.00 [-0.58, 2.57]
Brahim Belaid	0.9889	0.9946	15	0.862	0.3218	42	15.2%	0.13 [-0.39, 0.64]
Chenze Li	11.36	1.393	77	9.86	1.11	133	15.4%	1.50 [1.14, 1.86]
Elzbieta Kalici´nska	2.5124	0.5219	28	1.8251	0.286	54	15.5%	0.69 [0.48, 0.90]
Miao Luo	11.74	7.47	201	7.01	2.04	817	14.0%	4.73 [3.69, 5.77]
Takahisa Mikami	28.07	13.81	806	21.65	9.2	2014	14.1%	6.42 [5.39, 7.45]
Tao Chen	12.7145	6.7585	113	8.0756	2.1693	161	13.3%	4.64 [3.35, 5.93]
**Total (95% CI)**			**1268**			**3380**	**100.0%**	**2.67 [1.43, 3.90]**

Heterogeneity: Tau² = 2.53; Chi² = 210.72, df = 6 (P < 0.00001); I² = 97%
Test for overall effect: Z = 4.24 (P < 0.0001)

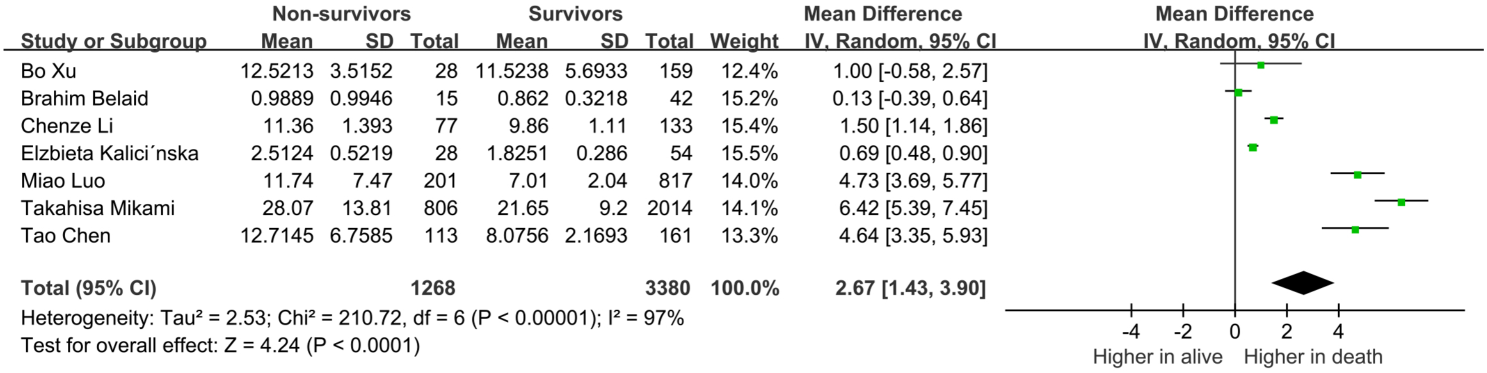

D

Study or Subgroup	Non-survivors Mean	SD	Total	Survivors Mean	SD	Total	Weight	Mean Difference IV, Random, 95% CI
Chenze Li	26.4136	7.7553	77	19.92	5.25	133	22.7%	6.49 [4.55, 8.44]
Fengqin Zhang	33.9621	38.9462	7	10.686	7.279	27	11.2%	23.28 [-5.71, 52.26]
Miao Luo	35.3857	29.5918	201	13.1085	7.9461	817	22.4%	22.28 [18.15, 26.40]
Takahisa Mikami	70.7073	55.4012	806	37.2607	24.0356	2014	22.4%	33.45 [29.48, 37.41]
Tao Chen	40.329	40.1002	113	13.2263	9.2758	161	21.3%	27.10 [19.57, 34.63]
**Total (95% CI)**			**1204**			**3152**	**100.0%**	**22.33 [8.75, 35.92]**

Heterogeneity: Tau² = 210.54; Chi² = 176.83, df = 4 (P < 0.00001); I² = 98%
Test for overall effect: Z = 3.22 (P = 0.001)

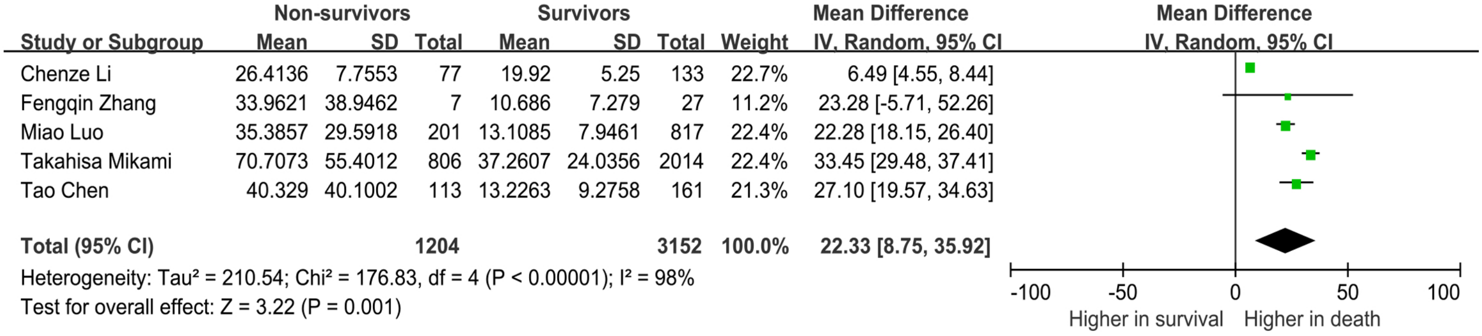

Supplementary Figure S5: Forest plot for the alive and death groups.

The serum levels of IL-2R in the groups of alive and death (A).

The serum levels of IL-10 in the groups of alive and death (B).

The serum levels of TNF-α in the groups of alive and death (C).

The serum levels of IL-8 in the groups of alive and death (D)
