# Supplementary material for: Increased Circulating Cytokines Have a Role in COVID-19 Severity and Death With a More Pronounced Effect in Males: A Systematic Review and Meta-Analysis
Source: Front Pharmacol. 2022 Feb 14;13:802228. doi: 10.3389/fphar.2022.802228 (PMC8883392; doi:10.3389/fphar.2022.802228)
Supplement: Supplementary file 2 [file Image6.PDF]

A

Study or Subgroup	Severe Mean	Severe SD	Severe Total	Mild Mean	Mild SD	Mild Total	Weight	Mean Difference IV, Fixed, 95% CI
Ai-Ping Yang	47.9103	95.749	24	21.6858	37.6312	69	0.0%	26.22 [-13.10, 65.55]
Bo Xu	5.1228	0.4838	107	5.0093	0.234	80	99.6%	0.11 [0.01, 0.22]
Shaohua Li	11.5088	9.6473	26	7.6773	1.4572	43	0.1%	3.83 [0.10, 7.57]
Yang Liu	7.5509	5.1376	30	6.8066	3.9028	46	0.2%	0.74 [-1.41, 2.90]
Ying Chi	13.6928	5.9865	8	15.7335	13.4878	58	0.0%	-2.04 [-7.45, 3.37]
**Total (95% CI)**			**195**			**296**	**100.0%**	**0.12 [0.01, 0.22]**

Heterogeneity: $Chi^2 = 6.44$, df = 4 (P = 0.17); $I^2 = 38\%$
Test for overall effect: Z = 2.19 (P = 0.03)

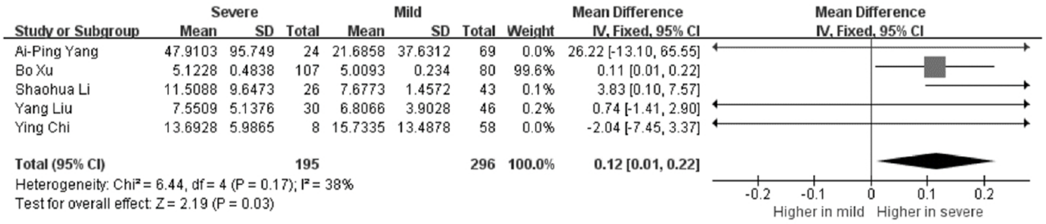

B

Study or Subgroup	Severe Mean	Severe SD	Severe Total	Mild Mean	Mild SD	Mild Total	Weight	Mean Difference IV, Fixed, 95% CI
Ai-Ping Yang	1.8963	2.3248	24	3.1506	5.0049	69	0.2%	-1.25 [-2.76, 0.25]
Changsong Wang	1.0411	0.7967	12	0.689	0.7207	33	1.5%	0.35 [-0.16, 0.87]
Huan Han	3.6087	0.4217	60	3.3752	0.6985	42	7.0%	0.23 [-0.00, 0.47]
Suxin Wan	1.83	0.185	21	1.69	0.07	102	61.2%	0.14 [0.06, 0.22]
Xiaohong Yuan	2.9081	1.4319	29	2.9431	1.3488	32	0.8%	-0.03 [-0.73, 0.66]
Yanli Wang	3.8395	2.2695	35	2.8542	1.0899	8	0.3%	0.99 [-0.08, 2.05]
Yi Li	2.641	0.9066	77	2.7475	0.9868	48	3.3%	-0.11 [-0.45, 0.24]
Ying Chi	9.192	2.8771	8	9.309	2.9956	58	0.1%	-0.12 [-2.25, 2.02]
Yingjie Wu	0.181	0.3233	39	0.2756	0.2949	32	19.0%	-0.09 [-0.24, 0.05]
Zhe Zhu	1.9936	1.1948	16	1.9544	0.8412	111	1.1%	0.04 [-0.57, 0.65]
Zhihua Lv	3.5119	1.7552	239	4.61	12.11	115	0.1%	-1.10 [-3.32, 1.13]
Zhongliang Wang	2.1618	0.3307	7	1.9749	0.3474	36	5.4%	0.19 [-0.08, 0.46]
**Total (95% CI)**			**567**			**686**	**100.0%**	**0.10 [0.03, 0.16]**

Heterogeneity: $Chi^2 = 18.98$, df = 11 (P = 0.06); $I^2 = 42\%$
Test for overall effect: Z = 3.01 (P = 0.003)

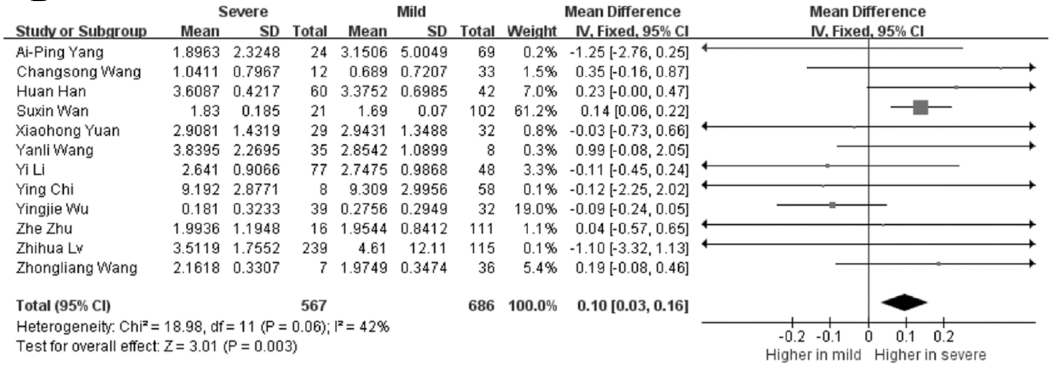

C

Study or Subgroup	Severe Mean	Severe SD	Severe Total	Mild Mean	Mild SD	Mild Total	Weight	Mean Difference IV, Random, 95% CI
Ai-Ping Yang	1,484.956	3,135.389	24	170.9148	343.981	69	0.0%	1314.04 [57.02, 2571.06]
Chenze Li	22.33	5.88	210	9.04	1.367	754	16.0%	13.29 [12.49, 14.09]
Chuan Qin	19.417	12.7411	286	14.5779	9.0489	166	15.1%	4.84 [2.82, 6.86]
Guang Chen	33.83	23.11	11	17.33	20.88	10	2.3%	16.50 [-2.31, 35.31]
Li-Da Chen	12.0336	2.2783	25	8.9	1.32	69	16.0%	3.13 [2.19, 4.08]
Shaohua Li	13.4933	3.5295	26	8.2254	3.0678	43	15.5%	5.27 [3.63, 6.91]
Wei Zhu	12.6974	2.5182	431	8.8516	1.4969	675	16.2%	3.85 [3.58, 4.11]
Xia Xu	49.0234	179.6116	41	7.5958	4.2825	47	0.3%	41.43 [-13.56, 96.42]
Yang Liu	50.7	89.33	30	10.52	9.95	46	0.9%	40.18 [8.09, 72.27]
Ying Chi	31.9154	30.7724	8	32.4792	25.1831	58	1.7%	-0.56 [-22.85, 21.72]
Zirui Meng	5.8301	1.4704	27	3.8409	1.0105	71	16.1%	1.99 [1.39, 2.59]
**Total (95% CI)**			**1119**			**2008**	**100.0%**	**5.96 [2.91, 9.02]**

Heterogeneity: $Tau^2 = 14.97$; $Chi^2 = 571.54$, df = 10 (P < 0.00001); $I^2 = 98\%$
Test for overall effect: Z = 3.83 (P = 0.0001)

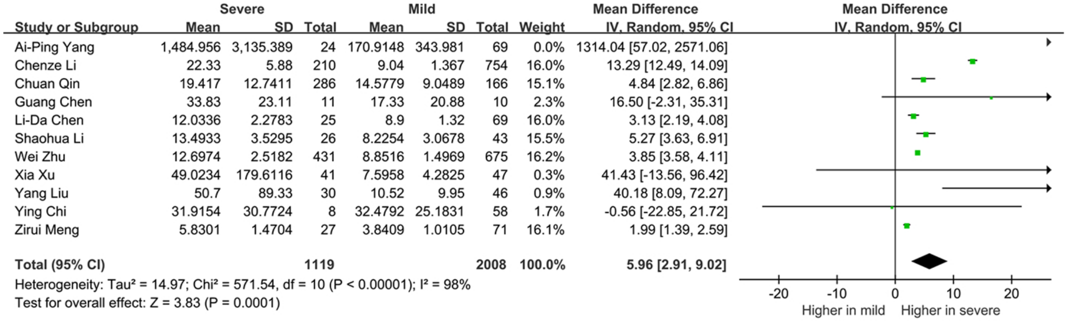

D

Study or Subgroup	Severe Mean	Severe SD	Severe Total	Mild Mean	Mild SD	Mild Total	Weight	Mean Difference IV, Fixed, 95% CI
Ai-Ping Yang	4.6011	7.7308	24	4.1533	7.6701	69	0.0%	0.45 [-3.14, 4.03]
Huizheng Zhang	17.0413	14.1024	14	9.8338	15.2127	29	0.0%	7.21 [-2.02, 16.44]
Suxin Wan	1.16	0.057	21	1.095	0.0226	102	100.0%	0.06 [0.04, 0.09]
Ying Chi	19.8181	8.3096	8	16.072	6.2924	58	0.0%	3.75 [-2.24, 9.73]
**Total (95% CI)**			**67**			**258**	**100.0%**	**0.07 [0.04, 0.09]**

Heterogeneity: $Chi^2 = 3.80$, df = 3 (P = 0.28); $I^2 = 21\%$
Test for overall effect: Z = 5.15 (P < 0.00001)

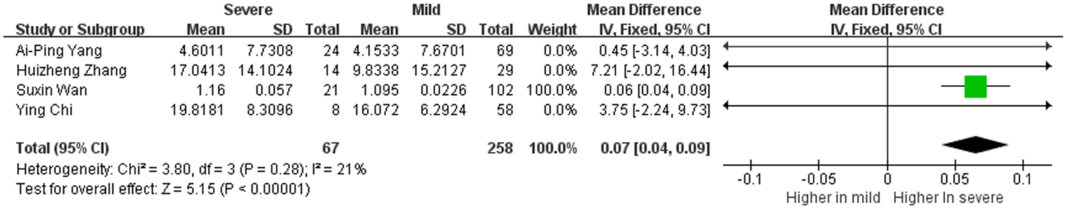

Supplementary Figure S6: Forest plot for the severe and mild groups

The serum levels of IL-1β in the groups of severe and mild(A)

The serum levels of IL-4 in the groups of severe and mild(B)

The serum levels of IL-8 in the groups of severe and mild(C)

The serum levels of IL-17 in the groups of severe and mild(D)
