# Supplementary material for: Increased Circulating Cytokines Have a Role in COVID-19 Severity and Death With a More Pronounced Effect in Males: A Systematic Review and Meta-Analysis
Source: Front Pharmacol. 2022 Feb 14;13:802228. doi: 10.3389/fphar.2022.802228 (PMC8883392; doi:10.3389/fphar.2022.802228)
Supplement: Supplementary file 3 [file Image4.PDF]

A

Study or Subgroup	Severe Mean	Severe SD	Severe Total	Mild Mean	Mild SD	Mild Total	Weight	Mean Difference IV, Random, 95% CI
Chenze Li	979.55	159.77	208	401.3	54.39	749	12.6%	578.25 [556.19, 600.31]
Chuan Qin	809.8828	452.8668	286	666.6957	291.2862	166	12.3%	143.19 [74.50, 211.88]
Guang Chen	1,191.33	404.44	11	405.83	109.25	10	10.0%	785.50 [537.09, 1033.91]
Li-Da Chen	723.55	126.51	25	390.66	77.18	69	12.4%	332.89 [280.06, 385.72]
Wei Zhu	591.759	87.2982	431	449.757	59.5462	675	12.6%	142.00 [132.62, 151.39]
Xia Xu	639.4711	764.4077	41	326.3944	211.0671	47	10.1%	313.08 [71.44, 554.71]
Yang Liu	1,716.5925	1,470.4358	30	828.0301	767.5512	46	5.3%	888.56 [317.54, 1459.58]
Ying Chi	181.6867	34.7574	8	189.2751	78.0534	58	12.5%	-7.59 [-38.95, 23.77]
Zhilin Zeng	906.9088	435.1493	224	686.6887	326.0635	93	12.2%	220.22 [132.82, 307.62]
**Total (95% CI)**			**1264**			**1913**	**100.0%**	**332.46 [159.91, 505.00]**

Heterogeneity: Tau² = 61586.27; Chi² = 1498.93, df = 8 (P < 0.00001); I² = 99%
Test for overall effect: Z = 3.78 (P = 0.0002)

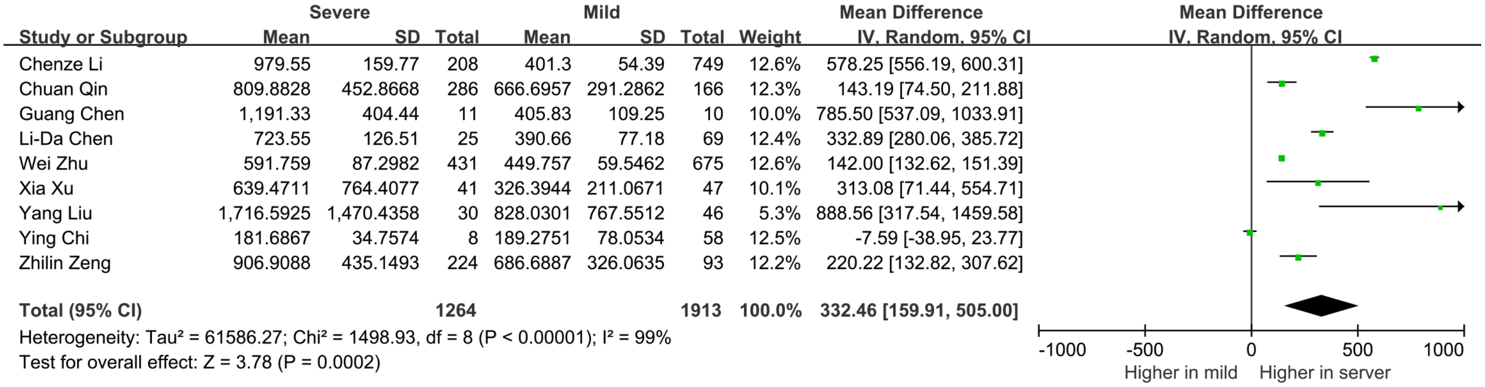

B

Study or Subgroup	Severe Mean	Severe SD	Severe Total	Mild Mean	Mild SD	Mild Total	Weight	Mean Difference IV, Random, 95% CI
Ai-Ping Yang	15.9226	30.222	24	4.4542	7.6625	69	0.2%	11.47 [-0.76, 23.69]
Bo Xu	9.8358	7.7367	107	5.726	1.7062	80	4.9%	4.11 [2.60, 5.62]
Changsong Wang	12.55	13.49	12	5.29	1.93	33	0.4%	7.26 [-0.40, 14.92]
Chuan Qin	7.63	4.66	286	5.7023	1.4957	166	7.5%	1.93 [1.34, 2.51]
Feng Gao	4.8916	1.0294	19	2.3222	0.5987	102	7.7%	2.57 [2.09, 3.05]
Guang Chen	10.76	1.55	11	6.06	2.37	10	4.3%	4.70 [2.97, 6.43]
Huan Han	6.5285	4.8159	60	5.2734	1.1898	42	5.5%	1.26 [-0.02, 2.53]
Huizheng Zhang	5.67	2.58	14	2.56	1.79	29	4.9%	3.11 [1.61, 4.61]
Susu He	4.93	6.13	33	3.49	2.13	60	3.4%	1.44 [-0.72, 3.60]
Suxin Wan	4.59	0.378	21	2.464	0.085	102	8.2%	2.13 [1.96, 2.29]
Tao Chen	13.7848	8.1102	113	6.1941	2.5434	161	4.8%	7.59 [6.04, 9.14]
Tao Liu	5.73	16.25	66	3.2	0.01	11	1.5%	2.53 [-1.39, 6.45]
Xiaohong Yuan	4.8013	1.9574	29	4.2369	1.4555	32	6.7%	0.56 [-0.31, 1.44]
Yang Liu	7.8868	4.8496	30	5.3082	0.6658	46	4.3%	2.58 [0.83, 4.32]
Yanli Wang	43.4037	92.5585	35	4.6085	1.801	8	0.0%	38.80 [8.11, 69.48]
Yi Li	4.6173	2.0398	77	3.7193	1.0954	48	7.6%	0.90 [0.35, 1.45]
Yi Zheng	7.3267	3.6805	15	5.8242	4.5655	19	2.5%	1.50 [-1.27, 4.27]
Ying Chi	11.4083	5.9776	8	9.4976	6.4477	58	1.2%	1.91 [-2.55, 6.37]
Yingjie Wu	6.469	5.6416	39	2.6569	2.5221	32	3.8%	3.81 [1.84, 5.79]
Zhe Zhu	6.9316	6.3238	16	3.2918	1.8177	111	2.1%	3.64 [0.52, 6.76]
Zhihua Lv	8.2772	9.3509	239	6.5	4.84	115	5.0%	1.78 [0.30, 3.26]
Zhilin Zeng	7.5303	4.6808	167	5.6338	1.3555	93	7.0%	1.90 [1.13, 2.66]
Zhongliang Wang	7.6379	6.7237	7	4.26	1.5171	36	1.0%	3.38 [-1.63, 8.38]
Zirui Meng	4.713	3.3132	27	1.1687	0.4484	71	5.6%	3.54 [2.29, 4.80]
**Total (95% CI)**			**1455**			**1534**	**100.0%**	**2.61 [2.08, 3.13]**

Heterogeneity: Tau² = 0.86; Chi² = 121.44, df = 23 (P < 0.00001); I² = 81%
Test for overall effect: Z = 9.77 (P < 0.00001)

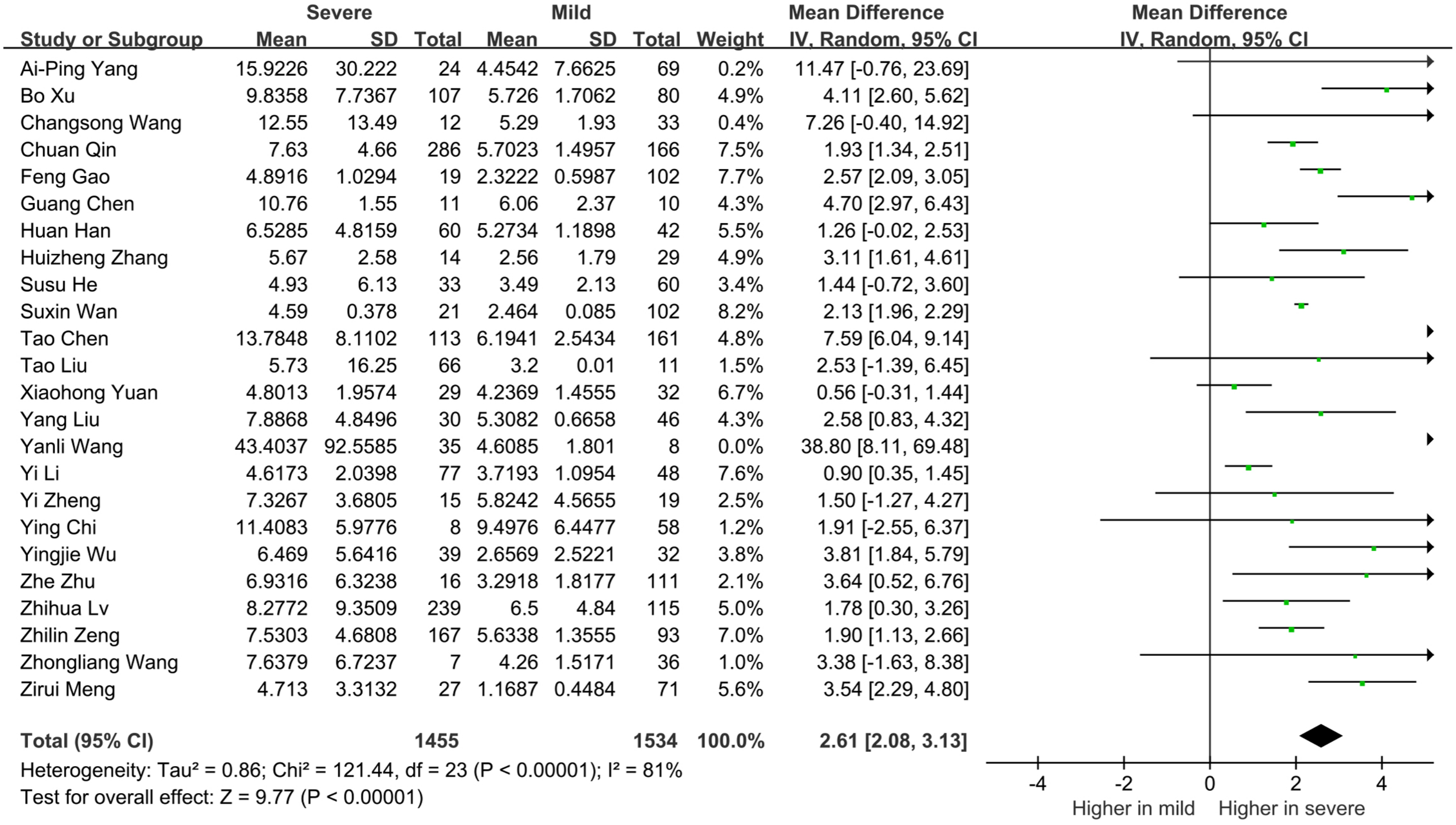

Supplementary Figure S4: Forest plot for the severe and mild groups.

The serum levels of IL-2R in the groups of mild and severe (A).

The serum levels of IL-10 in the groups of mild and severe (B).
