# Supplementary figures and images for: Increased Circulating Cytokines Have a Role in COVID-19 Severity and Death With a More Pronounced Effect in Males: A Systematic Review and Meta-Analysis
Source: Front Pharmacol. 2022 Feb 14;13:802228. doi: 10.3389/fphar.2022.802228 (PMC8883392; doi:10.3389/fphar.2022.802228)

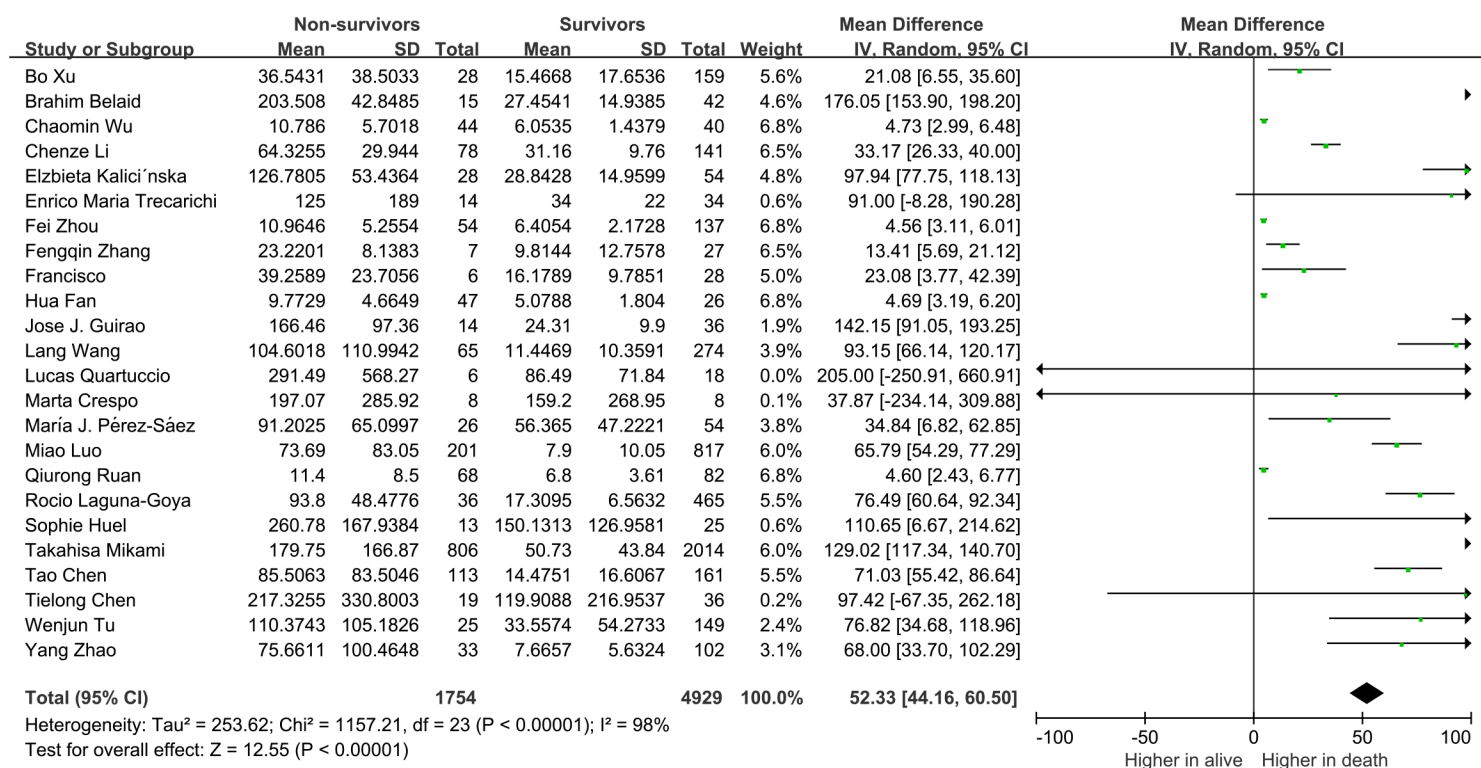

**Supplementary Figure S2:** The serum levels of IL-6 in the groups of alive and death

Supplement: Supplementary file 4 [file Image2.PDF]

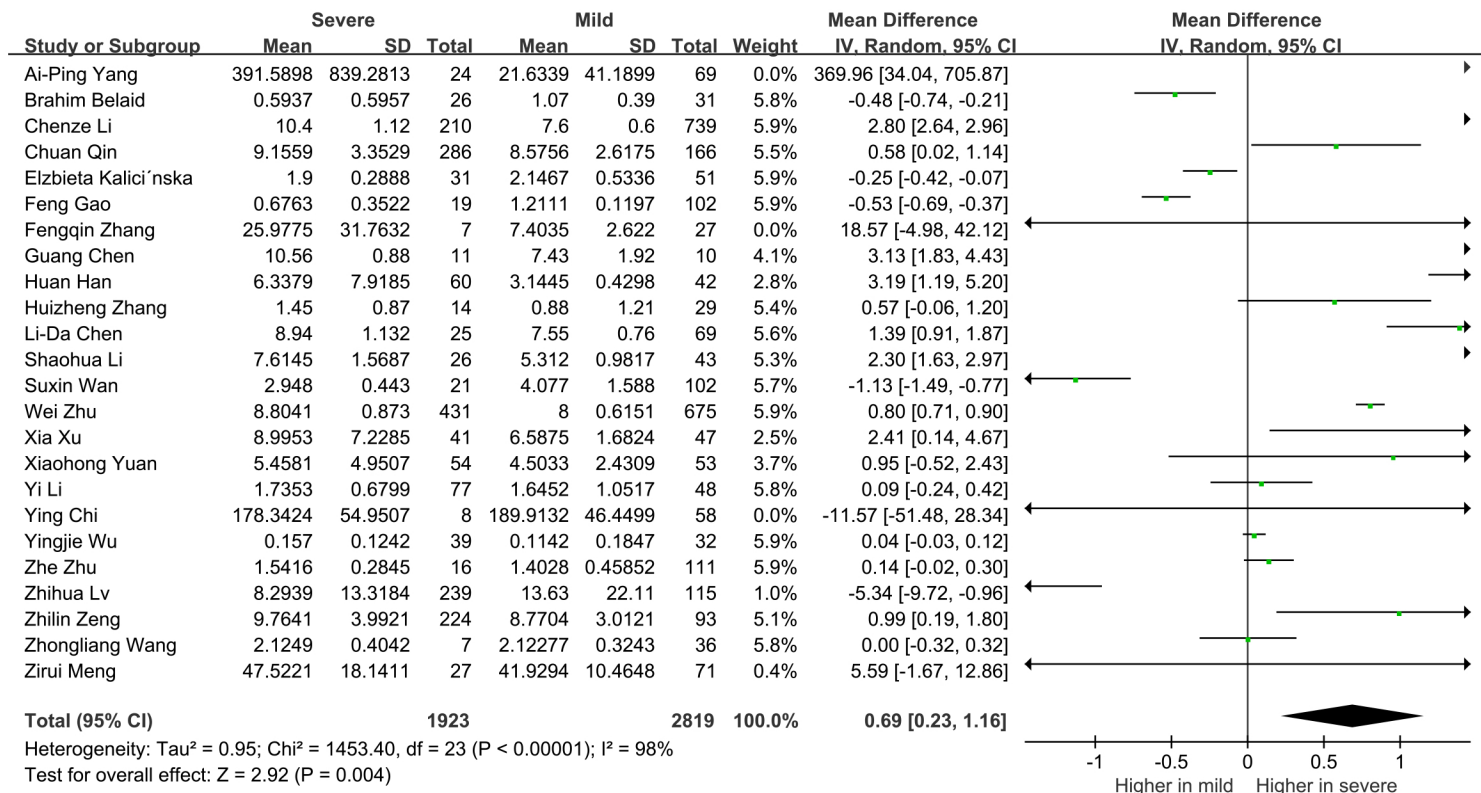

**Supplementary Figure S3:** The serum levels of TNF- $\alpha$  in the groups of mild and severe

Supplement: Supplementary file 5 [file Image3.PDF]

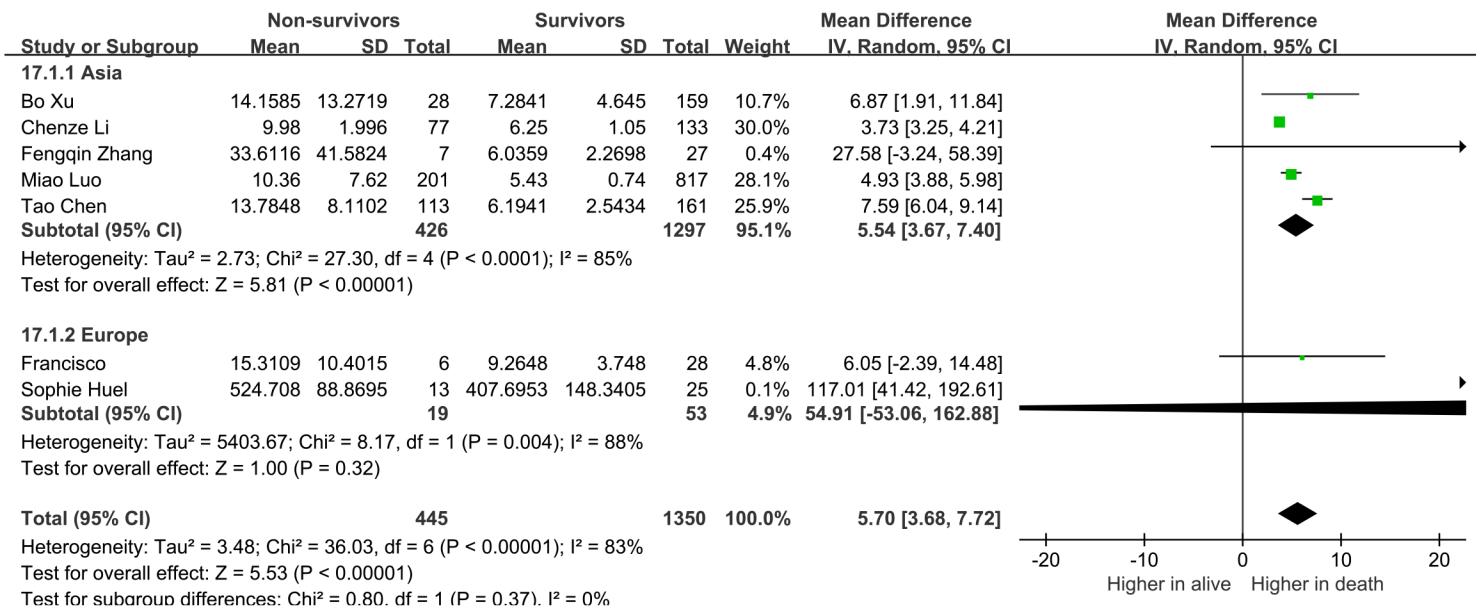

**Supplementary Figure S7:** The serum levels of IL-10 in the different continent of alive and death

Supplement: Supplementary file 6 [file Image7.PDF]

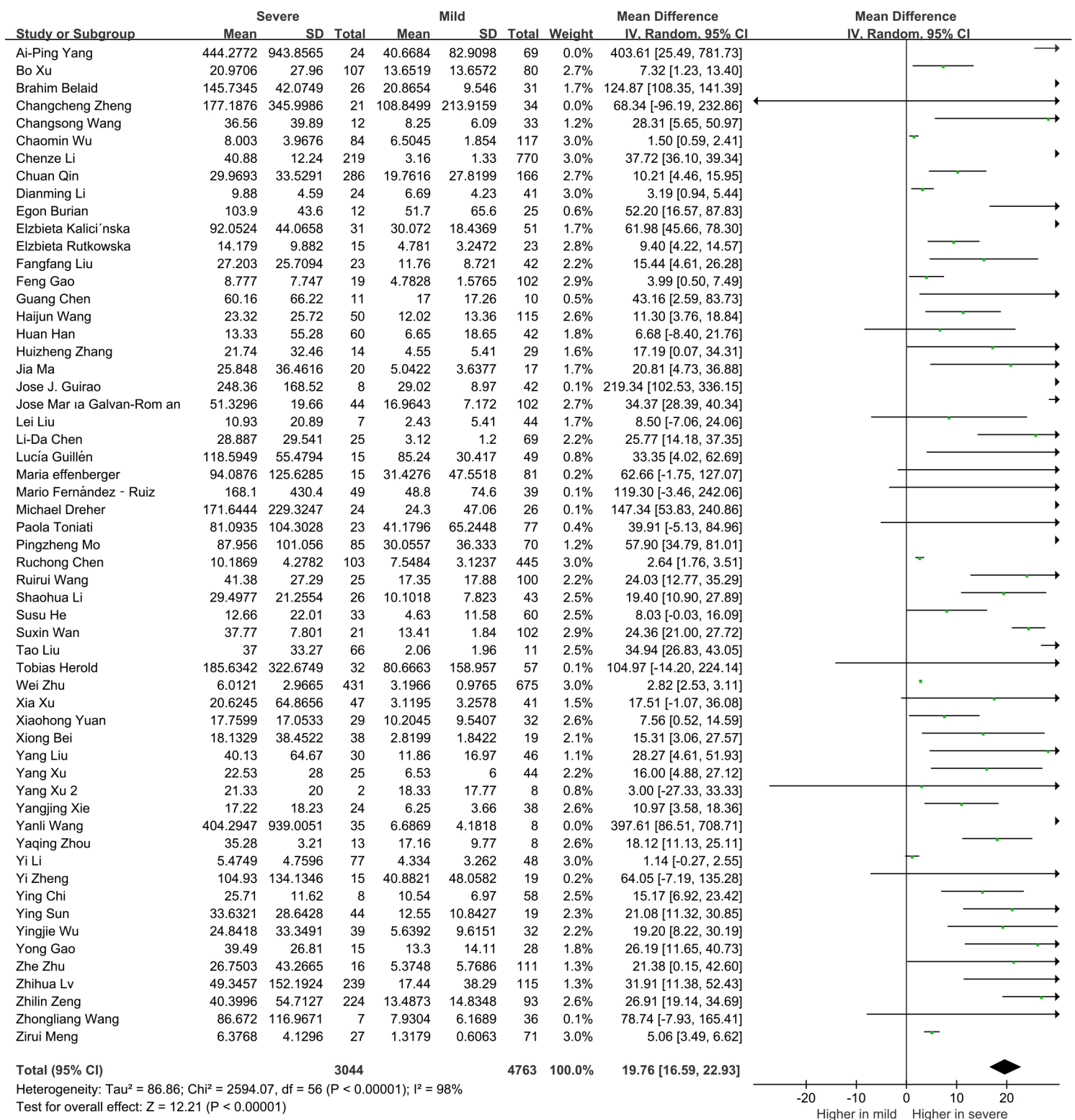

**Supplementary Figure S1:** The serum levels of IL-6 in the groups of mild and severe

Supplement: Supplementary file 8 [file Image1.PDF]
