# Supplementary material for: Increased Circulating Cytokines Have a Role in COVID-19 Severity and Death With a More Pronounced Effect in Males: A Systematic Review and Meta-Analysis
Source: Front Pharmacol. 2022 Feb 14;13:802228. doi: 10.3389/fphar.2022.802228 (PMC8883392; doi:10.3389/fphar.2022.802228)
Supplement: Supplementary file 7 [file DataSheet1.docx]

**Supplementary Table 1**. Basic clinical information of severe and mild patients in Europe and Asia

|  | Europe (n=803) | Asia (n=7107) | P value |
| --- | --- | --- | --- |
| Severe mean Age (year) | 62.9±6.7 | 59.03±11.5 | 0.2945 |
| Mild mean Age (year) | 59.56±6.2 | 49.8±11.6 | 0.02 |
| Severe/Mild | 256/547 | 2931/4176 |  |
| Male in mild group | 285/547 (52.1%) | 2141/4176 (51.3%) | 0.7139 |
| Male in severe group | 238/256 (92.9%) | 1485/2931 (50.7%) | ＜0.0001 |

**Supplementary Table 2.** Basic clinical information of death and alive patients in Europe and Asia

|  | Europe (n=719) | Asia (n=3560) | P value |
| --- | --- | --- | --- |
| Death mean Age (year) | 70.6±7.4 | 70.3±3.5 | 0.9246 |
| Alive mean Age (year) | 63.6±9.3 | 56.5±8.5 | 0.1086 |
| Death/Alive | 104/615 | 972/2588 |  |
| Male in alive group | 396/615 (64.4%) | 1294/2588 (50.0%) | 0.001 |
| Male in death group | 68/104 (65.4%) | 647/972 (66.6%) | 0.8087 |
